# Self-sampling for human papillomavirus testing among rural young women of KwaZulu-Natal, South Africa

**DOI:** 10.1186/s13104-017-3045-3

**Published:** 2017-12-06

**Authors:** J. N. Mbatha, H. N. Galapaththi-Arachchige, A. Mtshali, M. Taylor, P. D. Ndhlovu, E. F. Kjetland, M. F. D. Baay, Z. L. Mkhize-Kwitshana

**Affiliations:** 1 School Laboratory Medicine and Medical Science, University of KwaZulu-Natal, Durban, South Africa; 2 Department of Biomedical and Clinical Technology, Durban University of Technology, PO Box 1334, Durban, 4000 South Africa; 3 Norwegian Centre for Imported and Tropical Diseases, Department of Infectious Diseases, Oslo University Hospital, Oslo, Norway; 4 Faculty of Medicine, University of Oslo, Oslo, Norway; 5 Discipline of Public Health, Nelson R Mandela School of Medicine, University of KwaZulu-Natal, Durban, South Africa; 6 Claybrook Center, Imperial College London, London, UK; 7 Laboratory of Cancer Research and Clinical Oncology, University of Antwerp, Antwerp, Belgium; 8 Department of Biomedical Sciences, Mangosuthu University of Technology, Durban, South Africa

**Keywords:** Self-sampling devices, Self-collected samples, Cervico-vaginal lavage, Human papillomavirus

## Abstract

**Background:**

Cervical cancer is a major problem in women and it is important to find a suitable and acceptable screening method, especially among young in low-resource areas for future human papillomavirus (HPV) vaccine follow-up investigations. The study sought to test the acceptability of self-sampling as well as the suitability of the specimen collecting devices.

**Methods:**

Ninety-eight young women from rural KwaZulu-Natal were enrolled between March and July 2014. Collected genital specimens were transferred to colour indicator cards for HPV detection. Participants answered a questionnaire where they described their experiences with self-sampling. Samples were tested for high-risk HPV using GP5/6+ PCR.

**Results:**

Of the enrolled participants, 91 answered questionnaires and indicated that self-sampling was preferred by 51/91 (56%) women while 40/91 (44%) indicated preference for sampling by a doctor (p = 0.023). The majority, 64% were comfortable using a swab, 22% preferred a brush while 11% were comfortable with both devices. Of the 98 self-sampled specimens 61 were negative for HPV in both specimens while 37 were HPV-positive in either brush or swab. Of the 37, 26 (70%) were HPV-positive in both brush and swab (kappa = 0.743) and 11 (30%) were discordant.

**Conclusions:**

Self-sampling was acceptable to the majority of participants in this rural area. The Dacron swab was the preferred device, and can be used in combination with colour indicator cards for comfortable self-sampling, easy storage and transport of specimens plus detection.

## Background

The global burden of cervical cancer is estimated at 528,000 new cases and causes 266,000 annual deaths [1]. Eighty-five percent of the cervical cancer burden occurs in less developed regions such as sub-Saharan and developing countries [1]. Cervical cancer is the 5th most common cancer worldwide and the third most common in females [2]. The cervical cancer incidence is estimated at 31.5 cases per 100,000 people in South Africa and 11.5 per 100,000 in Europe with a mortality rate of 17.9 cases per 100,000 people in South Africa [1, 2]. South Africa is a middle-income country, with a large proportion of its population living in low resource rural areas. In these poor communities, cervical cancer screening strategies and reporting have not been successfully implemented [3]. Even when cervical cancer screening is offered in the local clinics, 87% of women do not participate as the women are unaware of the availability of services, scared of the procedure or cite religious and cultural reasons for not participating [4–6]. Cervical cancer screening may be improved by use of easily accessible testing for people from all socioeconomic levels. Some have recommended human papillomavirus (HPV)-based detection on self-sampled material to screen for cervical cancer [7].

A variety of sampling devices can be used for HPV detection for example Dacron swabs, cotton wool tipped swabs, cytobrushes and vaginal washings (lavage), either by self-collection or collected by a health worker. Studies in America, showed an 80% agreement between physician-directed swabs and self-collected vaginal tampons [8]. An excellent HPV detection agreement was reported between self-collected and physician collected cervico/vulvo-vaginal specimens in transport media from women aged 18–25 years [9]. In a meta-analysis on self-collected vaginal samples, cotton swabs and Dacron swabs were found to have an overall sensitivity of 0.74 and specificity of 0.88 compared to clinician-collected samples [10], whilst another meta-analysis showed that self- and clinician-collected samples gave an overall equivalent result for detecting HPV DNA [11].

Self-collected samples for HPV testing might be a better approach in areas where cultural and programmatic barriers may limit the use of routine gynecologic procedures [12]. This self-sampling strategy may be important as an alternative for cervical cancer screening possibly increasing the participation in primary screening and follow-up. In addition, self-sampling may be used for future HPV vaccination follow-up investigations among rural young women residing in low resource areas.

The main objectives of the study were to (i) assess the acceptability of self-sampling among young rural women, (ii) to evaluate the optimal sampling device, and (iii) investigate the use of Flinders Technology Associates (FTA) cartridge indicator cards for safe storage and transport of DNA for hr-HPV detection.

## Methods

### Study design and study area

The study had a cross-sectional design and was nested in a larger main clinical study. The latter was investigating Female Genital Schistosomiasis (FGS) and other sexually transmitted infections including HPV [13]. HPV was included because like in FGS, HPV is linked to cervical lesions. Kwa-Zulu-Natal has a subtropical climate with high humidity and generally warm temperature with high rainfall [14]. The study was carried out in the coastal areas which have a humid and hot climate with a lot of rainfall [14]. The study area has a population size of 630,464 [15]. A proportion of the population in this district was reported to have no access to piped water (19%) and 7.1 had no access to waste disposal system and 5.8% had no flush toilets [16, 17]. Rural Health Care facilities are situated in mountainous areas that are difficult to access resulting in the influx of people in urban clinics [16].

### Study participants

Female participants aged 16 years and above were recruited from randomly selected high schools from the rural district in the northern coastal region of KwaZulu-Natal, South Africa. This district has minimal infrastructure; much of the population lives in informal settlements and has little sanitation and other infrastructure. Young women were targeted for this study because of the assumption that they are sexually active and would provide enough HPV positive samples to be able to compare the sampling devices used.

**Fig. 1:**
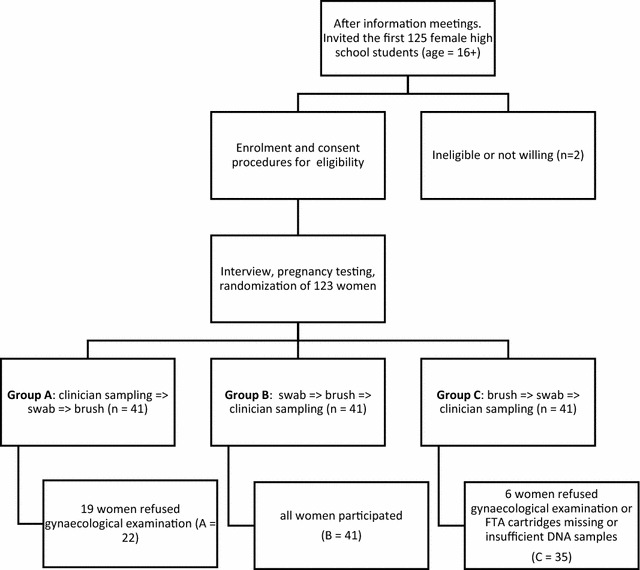
Number of participants recruited, enrolled and group randomisation. Numbers on the flow-chart indicate the number of participants at each stage of enrolment and randomisation of participants

The study population consisted of young women, recruited from high schools from a rural area in the northern coastal region of KwaZulu-Natal, South Africa. Inclusion criteria for the study were (1) attending the largest (more than 300 pupils) government high schools situated more than 10 kilometres from the coastline and below 300 m above sea level, (2) provided written informed consent, (3) sexually active, (4) not pregnant (5) above 16 years of age and (6) prepared to undergo self-sampling. Female participants were recruited from randomly selected high schools. Enrolment of participants into the study and group allocation was carried out following an outline in Fig. 1.

### Data collection

Trained research assistants interviewed the study participants in their local language, isiZulu. For sample collection each participant was given verbal, written and graphical instructions to follow when collecting a sample. The self-sampling devices, the Dacron swab-EZ (Becton–Dickinson, Erembodegem, Belgium) and the Viba-brush (Rovers Medical Devices B.V., Oss, The Netherlands), were alternated as first and second sampling device respectively per group. One group collected using a Dacron swab first while the other group of participants used a Viba brush first. After a sample was collected, it was transferred to a solid carrier, the indicator FTA elute card (Whatman Group, Kent, UK), which changes color from purple to white when enough material has been transferred. Cards were kept at room temperature until they were processed.

### Description of the questionnaire

After the sample collection, participants self-administered a questionnaire on pain experienced and their preferred specimen-collection method. The reasons for their preferences were asked using open ended questions. The questionnaire was completed confidentially in the waiting room area of the research site clinic.

### DNA extraction

Deoxyribonucleic acid obtained using swabs and Viba-brushes was extracted from FTA cards following the FTA elution method described previously [18]. Briefly, 3 mm punches were obtained using a sterile perforator (3 mm Harris Unicore device, Whatman). The punches were washed once with sterile distilled water. The DNA was eluted in 50 µl of sterile distilled water, by heating at 95 °C for 30 min. Extracted DNA was transferred to a clean tube, and kept at − 20 °C until analysis.

### High-risk human papillomavirus detection

Human papillomavirus was detected by GP5+/6+ HPV PCR [19] followed by an enzyme immunoassay method (EIA) [20] using a cocktail mix of high risk probes containing the hr-HPV types 16; 18; 26; 31; 33; 35; 39; 45; 51; 52; 53; 56; 58; 59; 66; 68; 73; and 82 (Whitehead Scientific, South Africa). The PCR mix consisted of nuclease free water, 10× PCR buffer with MgCl_2_, 25 mMol MgCl_2_, 2.5 µM GP5+ and 2.5 µM GP6+ primers, 10 mM nucleotides and 5 units taq polymerase [21]. For PCR, 10 µl of extracted DNA was added to 40 µl of PCR mix. These were run on 40 PCR cycles as follows: 5 min at 94 °C (Centigrade), 1 min at 94 °C, 2 min at 40 °C, 1 min 30 s at 72 °C and 10 min at 72 °C. At the end of 40 cycles the PCR products were kept at 4 °C until the EIA was performed. For the EIA, streptavidin coated plates (96 flat wells clear plates, Thermo scientific, Denmark) were used. The detection was performed using digoxin-labeled probes and monoclonal antidigoxin alkaline phosphatase antibody (A1054, Sigma, USA). A positive reaction was detected by the alkaline phosphatase substrate p-nitrophenyl phosphate (P4744, Sigma). The optical density (OD) was read at 405 nm using a spectrophotometer (Bio-Tek Instruments).

Six negative (DNA isolated from lung cancer cell line A549) and two positive controls (DNA isolated from cervical cancer cell lines HeLa and SiHa) were included in each run. The cut-off value for hr-HPV positivity was calculated as the mean plus three times the standard deviation of six negative controls in each plate. Samples with a ratio of 2 or more were regarded as positive.

### High-risk HPV genotyping

All the specimens from which hr-HPV was detected, as described above, were further analyzed to identify the genotypes. For genotyping, individual probes were used instead of a probe cocktail mix used for the EIA test. Individual probes for the most common hr-HPV genotypes in cervical cancer [22] were used in testing (16; 18; 26; 31; 33; 35; 39; 45; 51; 52; 53; 56; 58; 59; 66; 68; 73; and 82). The hr-HPV genotype was calculated following the formula of the mean OD plus three times the standard deviation of all samples in the plate. After excluding all the outliers, the final mean + 3SD was recorded and used as the cut off.

### Statistical analysis

The sample size was calculated based on the hypothesis that 50% young women will prefer a self-sampling procedure over the gynaecological examination and that 10% would prefer the gynaecological examination. In order to detect the difference with a significance level of 5% and power of 80% a sample size of 25 participants would be needed in each of the three groups for sample collection sequence and randomisation. In order to accommodate for uncertainties, and increase the power of the investigation, 125 participants were approached and of these, 98 were included in this study.

The data were captured and analyzed by the IBM SPSS Statistics 22 package.

The results on the preferred device were compared using 2 × 2 tables and Pearson Chi square test. The correlation of hr-HPV detection with Viba-brush and Dacron swab on the FTA card was determined using kappa statistics to determine the correlation beyond that expected by chance. A p value of < 0.05 was considered to imply a statistically significant difference between the variables. Kappa values were considered as poor (< 0.20), fair (0.21–0.4), moderate (0.41–0.60), good (0.61–0.80) and very good (0.81–1.00 and above) agreement, respectively [23]. Discordant results were defined as those that were HPV positive on one specimen type while negative on the other.

## Results

All 98 participants were females attending rural high schools (grades 10–12), and residing in low resource areas of the KZN province. The women were aged between 16 and 22 years (median age 18) and had an average of two sexual partners in their lifetime (SD = 1.227). In all, 79 (80.6%; 95% CI 71.9–87.2) had heard about sexually transmitted infections (STI) whereas 17 (17%; 95% CI 11.0–26.1) denied any knowledge of STI. All study participants did the self-collection of vaginal samples using the Viba-brush and the Dacron swab.

The questionnaire was completed by 91 participants (93%; 95% CI 86–96.5), whereas seven women did not return the completed questionnaire (reasons unknown). Of the 91 participants, 58 (63.7%; 95% CI 53.5–72.9) preferred a Dacron swab (p value < 0.001) whereas 20 (22%; 95% CI 14.7–31.5) preferred a brush for self-sampling. Ten (11%; 95% CI: 6.1–19.1) were comfortable with both and three (3.3%; 95% CI 0.8–10.02) did not indicate a preference. Only 5/91 (5.5%; 95% CI 2.4–12.2) women reported pain using a swab, whereas pain was reported by 25/91 (27.5%; 95% CI 19.4–37.4) using the brush. Of 91 participants 58 indicated a preference for self-sampling over collection with sampling by a doctor (OR = 2.44; 95% CI 1.62–3.68; p = 0.023). They cited reasons against clinician-sampling related to time, cost, discomfort, embarrassment and some were worried about meeting male doctors. However, 21 out of 91 (23%) participants who preferred self-sampling also gave reasons in favor of clinician-sampling, which included fear of inadequate self-sampling, trust in the doctor’s expertise in sample collection and disease diagnosis. Of the 91 participants who answered the questionnaire 52 (57.1%; 95% CI 46.9–66.0 linked clinicians’ expertise with better diagnosis and 8 (8.7%; 95% CI 4.5–16.4) indicated that they were scared to hurt themselves when performing self-sampling.

All 98 FTA cards had changed color, indicating that enough DNA had been transferred by the participants. Of the 98 participants, 37 were positive for hr-HPV, with 26 positive in both the brush and the swab and 11 were discordant. Of the 11 hr-HPV discordant results, three were negative in brush (positive in swab) and eight were negative in the swab (while positive in the brush) specimens. This resulted in a kappa value of 0.743, indicating good correlation of the HPV DNA detection between swab and brush specimens (Table 1). The sample size was not large enough to explore the differences in DNA yield between the sampling methods. The sequence of sampling had no significant effect on the HPV positivity of each sample type, however, this could represent a type 2 error.

**Table 1:** Hr-HPV detection in Dacron swab and Vibra-brush self collected specimens

	HPV positive		HPV negative		Total	p value	k value
	n	%	n	%			
Dacron swab	29	30 (0.21–0.39)	69	70	98	< 0.001	0.743
Viba brush	34	35 (0.26–0.45)	64	65	98		

At least one concordant genotype was found in 16 of the 26 participants that were hr-HPV positive in both brush and swab. In the brush we found a high number of hr-HPV genotype 39 (p = 0.011) and HPV 51 (p < 0.001) whereas in the swab type 66 was overrepresented (p = 0.046), for all other genotypes the prevalence was comparable in swab and brush specimens.

## Discussion

The Dacron swab was the self-sampling device preferred by our study population, possibly because it was found to be less painful. The study showed that cards can be kept at room temperature in an area such as ours which was remote, hot and humid. Young sexually active women were targeted for this study because of the assumption that they would provide enough HPV positive samples to be able to compare the sampling devices used.

The FTA cards have offered a useful collection and transportation tool for DNA test samples across a wide range of temperatures (22–45 °C) and humidity between 20 and 100% [24]. FTA card coupled with Viba-brush is suitable for HPV DNA testing [18] and has been used in other types of specimens and showed good stability for years when stored at room temperature [25, 26].

The fact that the swab specimens could be easily transferred to FTA cards may be beneficial for communities living in low resource areas provided the price is low. Self-sampling may be used to reach individuals who have difficulties reaching the health facilities, have religious reasons or are not comfortable with undergoing gynecological examination. Self-sampling for HPV screening has some advantages over collection by a health worker. These samples can be cost-effective, easy to collect and less demanding in terms of storage and transportation methods. For self-sampling to be effective it is necessary that the sampling tools or devices are user-friendly, that the targeted population is comfortable with using them, and that the laboratory test is able to detect the viral infection from such samples.

Reasons for discordant hr-HPV results between the preferred device and the brush could not be ascertained. Therefore, further research to work out the sensitivity and specificity of the Dacron swab specimen on FTA card for HPV test is necessary since this type of specimen was preferred. FTA cartridges and self-collected brush specimens have been tested previously for HPV molecular analysis [18].

Although self-sampling may seem easy and comfortable for women and is well-accepted by some [27, 28], it is met with less enthusiasm in other communities [29]. Currently there is no consensus on the preferred and most suitable sampling device. Previous reports have shown a high preference for home self-sampling due to religious and/or cultural beliefs and a feeling of embarrassment. The young and unmarried women have been found to be particularly hesitant about clinician-sampling or full gynecological examination [29, 30]. Although a statistically significant majority of our participants preferred self-sampling, a proportion of them were concerned regarding their ability to collect the sample correctly [30–32] and trusting the physician sampling [33]. This may indicate that training and information will be required to achieve a high uptake of self-sampling for female genital samples. Some participants indicated being scared of hurting themselves, a perception which if left unaddressed may lead to insufficient sample being obtained for optimal DNA analysis.

Successful implementation of self-sampling should be viewed in the light of the sampling tool used. In this study, two-thirds of the participants preferred using the swab thus suggesting that a swab can be used comfortably for self-collection of genital samples. Previous reports of self-sampling using swab specimens for HPV DNA analysis made use of transport media which can preserve the specimen [29] whereas some made use of self-collected cervico-vaginal lavage [28, 34]. However, with current local specimen transport system none of these methods would have been adequate for our rural setting with lack of good transport systems [16]. Poor transport system impacts on posting system in the rural areas. In our study we found that FTA elute cards yielded enough DNA for hr-HPV detection for both brush and swab specimens. This would eliminate the possibility of specimen leakage and the need for bigger size packaging. Furthermore, the FTA cards change color to indicate enough DNA has been obtained, which is an additional advantage as it will reassure women that their self-sampling procedure was correct thus giving confidence to women regarding their sampling technique.

Although we cannot preclude a type 2 error we found that neither device out-performed the other for hr-HPV detection. A good correlation was observed between the swab and the brush with regards to the number of positive and negative samples in this study, however, discordant results were found in hr-HPV genotypes in the specimens. Other reports have shown a higher prevalence of HPV detection from specimens collected in FTA cards than those in liquid medium from Costa Rica women involved in phase III vaccine trial [35]. In addition, DNA yield in FTA card collected specimens was 3.5 times more than those in liquid medium. Furthermore, previous reports have indicated a higher prevalence of certain HPV types in the vagina compared with the cervix in women younger than 50 years [36]. Variations in the actual sampling technique and the anatomical sites touched by the woman inside the vagina may contribute to the discordant results between self-sampling devices [36]. In the United States, the average length of vagina in premenopausal women not having undergone hysterectomy has been estimated at 9.2 cm [37] which may suggest that the self-sampled specimens are of lower vaginal tract origin.

The cytological tests (Pap smears or liquid based cytology) are currently the most widely used and available conventional test for cervical cancer screening [38, 39]. However, there has been a growing interest regarding use of HPV DNA detection and molecular techniques [40]. Cytology was reported to have low sensitivity and high specificity for cancer diagnosis when compared to HPV DNA detection, and primary HPV screening has recently been endorsed by scientific societies and regulatory agencies in the US and Europe for women [41]. Furthermore, HPV detection on self-collected samples has been introduced as a way to decrease non-participation in cervical cancer screening programs [42–44].

Whereas cervical cancer screening would normally aim at older women, we included young women for self-sampling to increase the chances of detecting HPV as infection is most common in this age group. It is not advisable to advocate HPV screening on young women since HPV infection will usually regress [45]. The HPV screening has high sensitivity and low specificity and positive results in young women may instigate unwarranted fear of imminent cervical cancer [46]. However young women are expected to have a higher HPV prevalence [47]. In order to compare the two sampling devices a likely high-endemic population was sought. Furthermore, given the high HIV prevalence in South Africa and in the KwaZulu-Natal province, young women are more likely to be prone to developing high grade lesions and cervical cancer. Women with HIV are more prone to HPV infection and were also reported to have a greater than 40-fold chance to develop squamous intraepithelial lesions [48].

Limitations in this study were the sample size, which due to financial constraints was kept at a (statistically acceptable) minimum and consistency in the sampling technique. For privacy reasons, we could not ascertain the consistency in the self-sampling technique between participants regarding the anatomical part of the genitalia each woman touched during sampling.

The HIV status was not included in the study since it was not a prevalence study but it was merely to assess FTA card in combination with the Dacron swab against a Viba brush for HPV analysis. However, given the high HIV prevalence in South Africa, and hence, the potential impact, it would have been interesting to know if HIV infection had any significant effect on participant acceptability for self-sampling.

## Conclusions

Self-sampling was accepted by our rural study participants and the Dacron swab was comfortable for self-collection of female genital samples. Use of the Dacron swab coupled with the FTA indicator cards showed that sufficient DNA was collected and preserved. This study showed that the method can be used for identifying HPV and is acceptable to rural women. However, we would not encourage use of self-sampling for HPV screening in young women because a positive HPV result may cause anxiety and fear that they have cancer. Further investigation is necessary involving older age groups from rural KZN province on the acceptance and usefulness of self-sampling. However, this study shows that self-sampling is accepted and feasible, and therefore, self-sampling collection of specimens for HPV analysis may be of value for HPV vaccine rollout follow-up investigations in KZN. There will also be a need for proper training of women on the technique self-sampling technique.

## Abbreviations

HPV: human papillomavirus
Hr-HPV: high risk human papillomavirus
DNA: deoxyribonucleic acid
EIA: enzyme immunoassay
PCR: polymerase chain reaction
