# Correction to: Self-sampling for human papillomavirus testing among rural young women of KwaZulu-Natal, South Africa

**DOI:** 10.1186/s13104-018-3252-6

**Published:** 2018-02-26

**Authors:** J. N. Mbatha, H. N. Galappaththi‑Arachchige, A. Mtshali, M. Taylor, P. D. Ndhlovu, E. F. Kjetland, M. F. D. Baay, Z. L. Mkhize‑Kwitshana

**Affiliations:** 1 School Laboratory Medicine and Medical Science, University of KwaZulu-Natal, Durban, South Africa; 2 Department of Biomedical and Clinical Technology, Durban University of Technology, PO Box 1334, Durban, 4000 South Africa; 3 Norwegian Centre for Imported and Tropical Diseases, Department of Infectious Diseases, Oslo University Hospital, Oslo, Norway; 4 Faculty of Medicine, University of Oslo, Oslo, Norway; 5 Discipline of Public Health, Nelson R Mandela School of Medicine, University of KwaZulu-Natal, Durban, South Africa; 6 Claybrook Center, Imperial College London, London, UK; 7 Laboratory of Cancer Research and Clinical Oncology, University of Antwerp, Antwerp, Belgium; 8 Department of Biomedical Sciences, Mangosuthu University of Technology, Durban, South Africa

## Correction to: BMC Res Notes (2017) 10:702 10.1186/s13104-017-3045-3

Following publication of the original article [1], one of the authors reported that his name had been spelled incorrectly. It should be Galappaththi-Arachchige, not Galapaththi-Arachchige.

The original article has been corrected.
